# An innovative HIV testing service using the internet: Anonymous urine delivery testing service at drugstores in Beijing, China

**DOI:** 10.1371/journal.pone.0192255

**Published:** 2018-02-22

**Authors:** Xiaoxia He, Guowu Liu, Dongyan Xia, Xia Feng, Yi Lv, Huanyi Cheng, Yuehua Wang, Hongyan Lu, Yan Jiang

**Affiliations:** 1 National HIV/HCV Reference Laboratory, National Center for AIDS/STD Control and Prevention, China CDC, Beijing, China; 2 Beijing Center for Disease Control and Prevention, Beijing, China; 3 Beijing Research Center for Preventive Medicine, Beijing, China; 4 Department of Clinical Laboratory, Beijing Youan Hospital, Capital Medical University, Beijing, China; China Medical University, CHINA

## Abstract

Innovative human immunodeficiency virus (HIV) testing services will be needed to achieve the first 90 (90% of HIV-positive persons aware of their infection status) of the 90-90-90 target in China. Here, we describe an internet-based urine delivery testing service delivered through three pilot drugstores in Beijing that send specimens to a designated laboratory for HIV. From May 2016 to January 2017, we provided 500 HIV urine-testing service packs for display at the drugstores, and a total of 430 (86.0%) urine specimens were mailed back. All of the 430 urine specimens were of good quality and were tested. 70 urine specimens were HIV positive, showing a 16.3% (70/430) positivity rate. A total of 94.3% (66/70) of the HIV-positive participants obtained their test results through the internet, and 69.7% (46/66) of these participants received follow-up care. A total of 40 out of 46 (87.0%) participants agreed to have their results confirmed by a blood test, and 39 out of 40 (97.5%) participants were confirmed as HIV-1 positive, including two individuals that were previously diagnosed. Lastly, 28 out of 37 (75.7%) of the study participants were referred to the hospital and provided free antiviral treatment. Our data indicate that this innovative HIV testing service is effective and play an important role in HIV testing and surveillance.

## Introduction

The first 90 of the 90-90-90 target outlined by the Joint United Nations Programme on HIV/AIDS (UNAIDS) is the most important. It states that, by 2020, 90% of all individuals living with human immunodeficiency virus (HIV) should know their HIV serostatus [1]. Access to HIV testing is not only a public health imperative but also a requirement for effective HIV prevention and care. However, access to HIV testing and counseling (HTC) remains inadequate, and many individuals, including those at high risk, do not know their serostatus [2].

Despite the fact that HIV testing services are widely available in China, including facility-based provider-initiated testing and counseling (PITC) and voluntary counseling and testing (VCT), it is estimated that only 68% of all individuals with HIV were aware of their serostatus in 2015 [3]. Furthermore, the HIV testing rate among men who have sex with men (MSM) remains low [3,4]. This low uptake may be due to fears of disclosing personal information or being subject to discrimination or to the disincentive of a long wait time for results. The Chinese government has recognized this, and it has charged public health agencies with improving support services through the use of the internet, microblogs, wechat, and HIV laboratory networks, to scale up HIV testing services [5]. Thus, many innovative HIV testing services have been initiated to protect the privacy of patients and improve the accessibility of these services.

An HIV testing service was begun in the United States [6] that requires users to collect a dried blood spot (DBS) on a card at home, mail it to a laboratory for analysis, and obtain the results by telephone a few days later. However, this type of testing service is not popular because many users find it difficult to obtain a sufficient blood specimen [7]. As an alternative, many studies have shown that urine specimens show good sensitivity and specificity when used for HIV-1 antibody testing. Urine collection is non-invasive and convenient, and the specimens are non-infectious [8–13]. Furthermore, China has well-coordinated HIV laboratory network and quality assurance systems [14] that ensure infected persons receive timely diagnosis and treatment.

With the increase in gay websites in China, the internet is a good platform for expanding HIV testing services [15–19]. Therefore, we developed an innovative service for internet-based HIV testing of urine specimens. Our objectives were to improve the willingness of a high-risk population to proactively test for HIV and to increase the accessibility of HIV testing services in this population. In this report, we describe an HIV testing service delivered through drugstores frequently used by MSM in Beijing, China.

## Methods

### Ethical considerations

All study relevant details including the collection of participant urine and blood samples were approved by the Ethics review committees of National Center for AIDS/STD Control and Prevention, China CDC (X131022302), and all performed in accordance with relevant guidelines and regulations. Provided informed consent within HIV urine-testing service package for participants who voluntarily obtained the service package and collected urine by themselves. The blood samples were collected from individuals who agreed to do confirmed test, and they all signed informed consent before collected their venous blood.

### Sample collection

**Fig 1:**
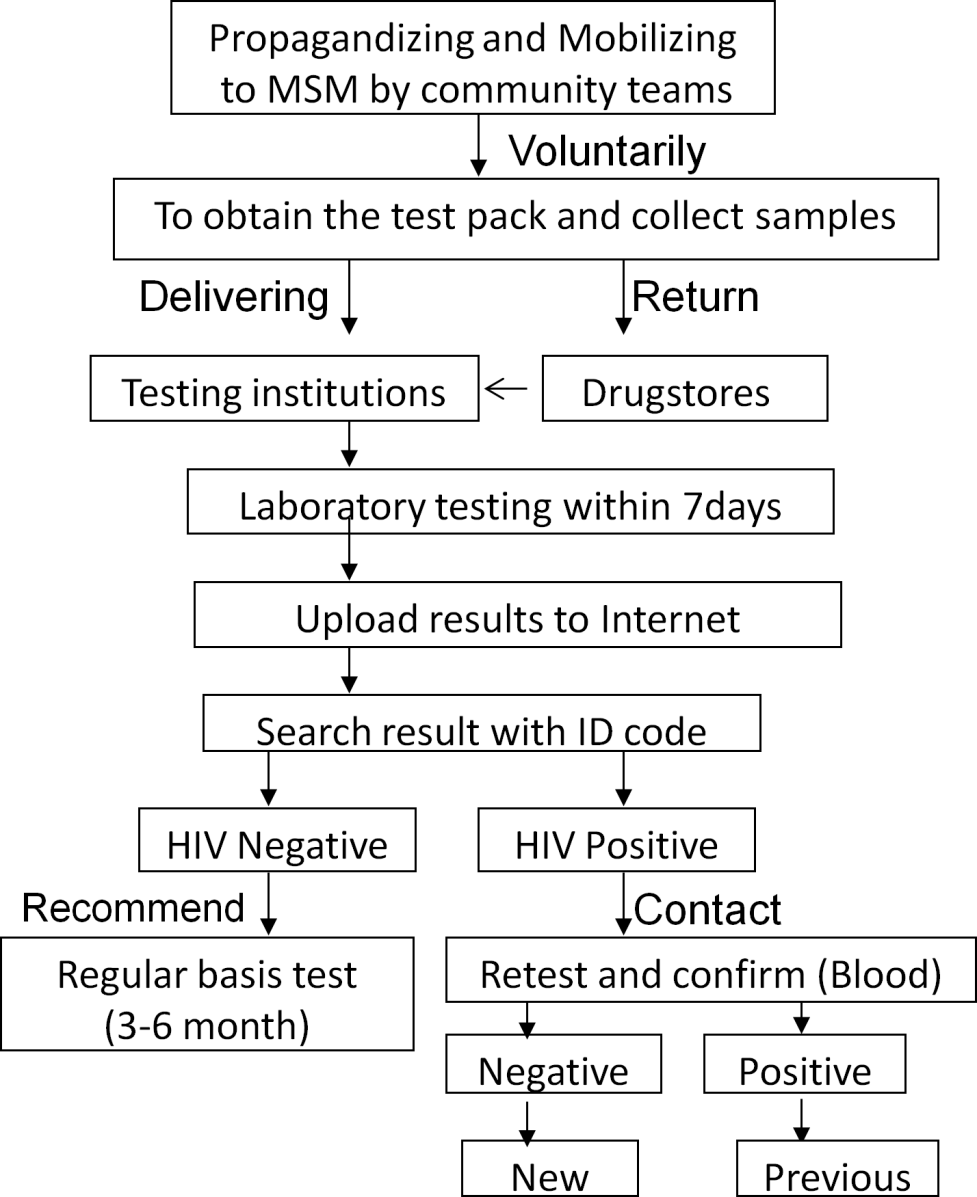
Outline of the service to test HIV antibody in urine by providing service package in drugstores.

The pilot study was conducted from May 2016 to January 2017 in three drugstores that were selected randomly from those that were adjacent to subways stops in busy districts of Beijing. The publicity campaign for this innovative HIV testing service, designed to reach MSM, was delivered by staff of MSM study teams through websites, online chat rooms, and offline partner introduction channels. Individuals who chose to participate voluntarily went to one of the three drugstores, obtained a urine-testing kit, and left their contact information (without name). Their pick-up time was registered by staff in the drugstores. Privately, each participant collected a urine sample and either mailed the specimen to the Clinical Laboratory at the Beijing Youan Hospital or returned it to the same drugstore who mailed it to the Clinical Laboratory. Instructions on these procedures, including information on sample collection, required urine quantity, sample mailing address, a unique sample identification code, and the website to retrieve results, could be found inside the urine test package require sample (Fig 1).

### Laboratory testing and results retrieval

After the designated laboratory received a urine specimen, the volume and properties of the specimen were determined. A urine HIV-1 antibody ELISA kit (Beijing JunHe Pharmaceuticals Co., Ltd., Beijing, China) was used to test eligible urine samples within 7 days after receipt of the specimen according to a strict protocol [20]. The test results were uploaded to a website (http://www.renaijiance.com/cui/pages/searchreport.aspx) that the participants could access to retrieve their results using the unique identification code on the label of each collection vessel.

### Support services

First, for the participants who tested HIV-1 antibody positive, we provided links with instructions to access local medical laboratories on the search results website. For those participants who voluntarily contacted one of the medical institutions to confirm their results, we provided a western blot diagnostic assay kit (MP Biomedicals) to test their venous blood specimens. All the confirmed HIV-infected participants were referred to the hospital and provided free antiviral treatment.

Second, we attempted to reach HIV-1 antibody-positive participants who did not contact the medical institution to accept support services. This information, which did not include names, was collected when each participant accepted a urine-testing service package. These participants were language motivated to accept the support services.

Finally, for the participants whose urine tested HIV-1 antibody negative, we recommended (on the website with test results) that they routinely test for HIV every 3 to 6 months.

## Results

### Sample collection

**Fig 2:**
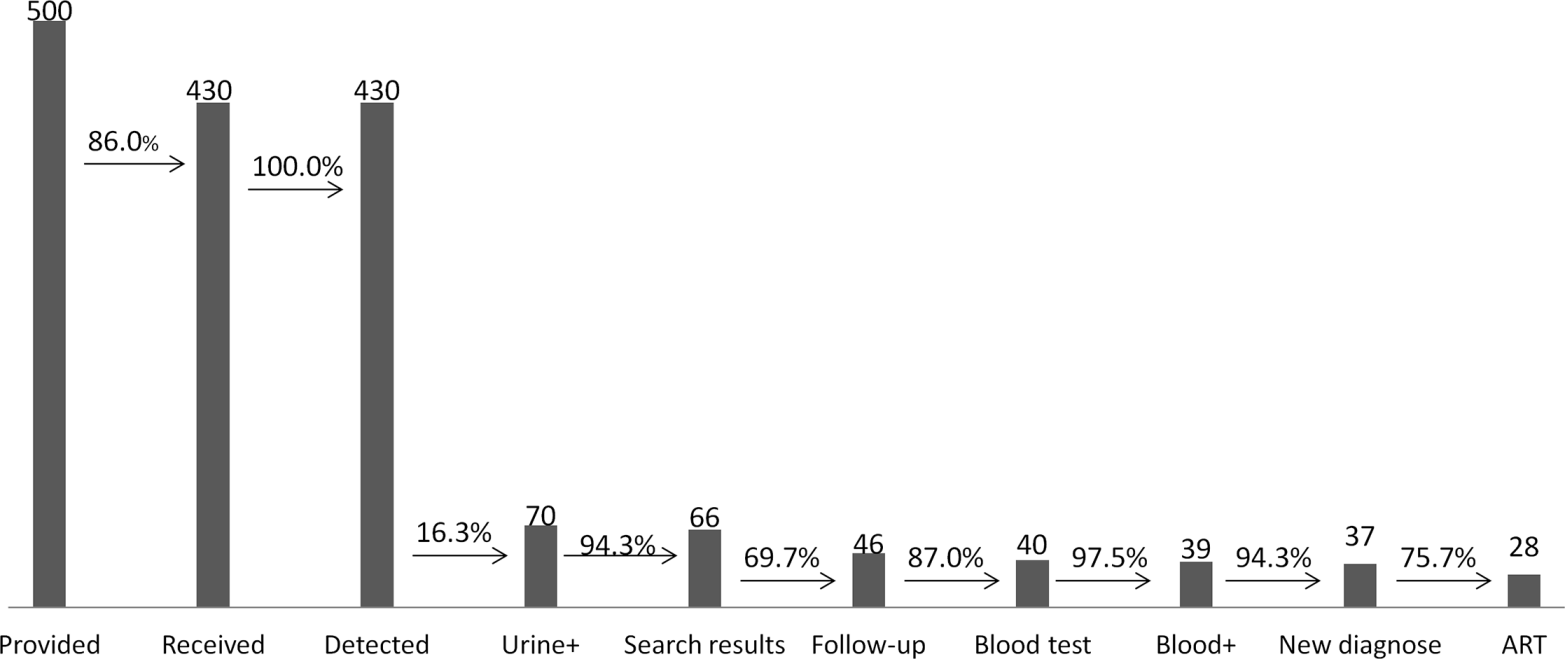
Results of the HIV testing service in 2016. The number at the top of the figure indicates the number of participants. The percentages indicate the proportion of the latter that account for the former. + indicates individuals who were found HIV positive. ART, antiretroviral treatment.

From May 2016 to January 2017, we provided 500 HIV urine-testing service packages to the three drugstores in Beijing, China. A total of 430 (86.0%) urine specimens were mailed to the designated testing laboratory (Fig 2).

### Laboratory testing

All of the 430 urine specimens were checked for quality and then tested for HIV-1 antibody at the Clinical Laboratory of Beijing Youan Hospital using a urine HIV-1 antibody ELISA kit. 70 out of the 430 urine specimens tested were determined HIV-1 antibody positive, showing an HIV-1 positivity rate of 16.3%. The remaining 360 (83.7%) urine specimens were HIV-1 antibody negative (Fig 2).

### Search results

Data from the results website showed that 66 of 70 (94.3%) HIV-1 antibody-positive participants and 232 of 360 (83.7%) HIV-1 antibody-negative participants searched for their test results by logging onto the website with a unique identification code. The number of times search results were viewed by participants in each group was 0 to 16 and 0 to 31, respectively (Fig 2, S1 File Data and S2 File Data).

### Support services

46 of 70 (65.7%) HIV-1 antibody-positive participants were followed, and 40 of 46 (87.0%) agreed to have their test results confirmed by western blot testing of their venous blood. A total of 39 out of 40 (97.5%) participants were confirmed HIV-1 antibody positive investigation. Of these 39 HIV-infected persons, 37 (94.9%) were newly diagnosed, and two (5.1%) were previously diagnosed. Lastly, 28 out of 37 (75.7%) newly diagnosed persons were referred to the hospital and provided free antiviral treatment (Fig 2, S3 File Data).

## Discussion

We developed an innovative internet-based HIV testing service in collaboration with three drugstores near subways in busy districts of Beijing. This service resulted in 86.0% (430/500) of participants who collected their urine tested for HIV-1 antibody, and, of these, 16.3% (70/430) were HIV-1 antibody positive. Of these 70 HIV-1 antibody positive participants, 66 (94.3%) learned of their HIV status by logging onto a results website, and 46 of 70 (65.7%) HIV-1-positive individuals were followed and received support services. In total, 40 of 46 (87.0%) individuals went to a diagnostic laboratory and provided a venous blood specimen for a western blot confirmatory test, and 39 out of 40 (97.5%) individuals were confirmed HIV infected, including 37 individuals who were newly diagnosed and two individuals who were previously diagnosed. Finally, 28 out of 37 (75.7%) HIV-1-infected individuals who were newly diagnosed were successfully referred to a hospital for free antiviral treatment. The above data indicate that this innovative testing service may greatly contribute to the acceptance of HIV testing, prevention, and treatment.

In this study, we selected urine specimens for collection, which was safe, convenient, and non-invasive, and these specimens demonstrated good sensitivity (94.34–100%) and specificity (95.39–100%) [13]. Our data showed that all of the urine specimens mailed to the laboratory were of sufficient quality for testing. In addition, 97.5% (39/40) of the participants whose urine samples were positive for HIV-1 antibody were confirmed HIV-1 antibody positive using venous blood, which indicated the suitability of using urine specimens for this anonymous HIV testing service [21].

Since 2015, HIV test kits for urine specimens have been provided in drugstores of Beijing and results have been reported by internet. This approach to testing has shown good acceptability [22]. However, there has been no way to contact individuals who are HIV-1 antibody positive but reluctant to contact a medical institution to accept support services. To fill this gap, in this study, we asked each participant to leave their personal information, including their gender and contact details (without name) and the time when they accepted the testing service package. This has allowed contact with and referral of HIV-positive individuals to the hospital for antiviral treatment. However, there were several participants unwilling to seek support services for fear of being discriminated against when they become aware of their HIV status. We will continue to seek better ways to address such discrimination.

In Beijing, the MSM population is the main group to be affected by HIV infection, with the rate of HIV infection positivity increasing from 1.4% in 2005 to 8.0% in 2015 [23,24]. There were a total of 21,886 new HIV cases in Beijing in 2016, and 90.1% of these cases had acquired HIV through sexual transmission; of these new HIV cases, 66.3% were MSM. However, the HIV testing rate in the MSM population remains low [4,25]. Studies have shown that MSM prefer to use the internet to socialize and access health information; thus, the internet is a good medium to reach and encourage the MSM population to seek HIV testing [19,26,27]. To achieve this, we combined online (gay websites and chat software) with offline (partner introductions) forums to encourage the MSM population to seek HIV testing. Our findings also showed that this innovative approach eliminated the shortcomings of traditional HIV testing models (VCT or PITC) such as those that require the disclosure of personal information and long wait times.

In Beijing, a total of 4,831,920 individuals were offered HIV antibody screening in 2016, and 3844 (0.080%) new HIV-1 positive cases were identified; 73.73% (2834/3844) were MSM. Among these new cases, 22.87% (879/3844) were identified through traditional VCT, 75.31% (2895/3844) were identified through traditional PITC and other models, and 1.82% (70/3844) were identified through our innovative model. Compared to the number of new cases identified through routine screening and VCT [0.080% (3844/4,831,920) and 4.53% (879/19387), respectively], the rate of new cases identified through our innovative model was 16.3% (70/430). This significant difference was attributed to the population targeted by our study; we focused on the MSM population, which is the main group infected with HIV in Beijing. These results suggest that we should pay more attention to key populations for the promotion of HIV antibody screening, because this approach is more cost-efficient. Although there were a few false positives in this study, because not all of the 70 individuals determined HIV-1 antibody positive by urine testing were confirmed positive by testing of venous blood specimens, this conclusion is still valid because of the good agreement between the results of urine and blood testing.

These contributions of the study were observed with only three pilot drugstores and 500 HIV tests kits provided. As this innovative HIV testing service is scaled up in the future, these contributions will increase. In addition, among the individuals living with HIV in Beijing during 2016, approximately 82% of them learned of their serostatus through a traditional detection model (PITC, VCT, and others). The remaining 18% did not know their HIV serostatus, and the penetration of traditional detection methods had already reached saturation. Moreover, with our innovative service, 94.3% (66/70) of individuals determined HIV-1 antibody positive by testing of urine specimens logged onto the study website to find their test results. The maximum number of times these results were viewed by HIV-1 antibody-positive and -negative participants was 16 and 31, respectively, the mental dynamic indicating their strong desire to know and extreme concern about their HIV serostatus. Given the above, there is an urgent need to develop innovative HIV testing services to achieve the first 90 of 90-90-90 target.

According to 2015 data from the Beijing Centers for Disease Control (unpublished), the HIV-1 antibody positivity rate in the MSM population as determined through VCT was 11.76% (890/7566). In contrast, the HIV positivity rate in the MSM population as determined through our innovative HIV testing service model in 2016 was 16.3% (70/430). This higher positive rate indicates the greater willingness of MSM to use an anonymous testing service to learn of the HIV status.

There are many individuals in China seeking kits available on the internet for rapid HIV self-testing of saliva, but the accuracy of these results is not guaranteed [28]. Our innovative anonymous testing service not only provided accurate test results but also promoted treatment referrals, contributing to both the first and second 90 (90% linked to anti-retroviral therapy “ART”) of the 90-90-90 target.

## Conclusions

In conclusion, we developed a unique and innovative HIV testing service. It was based on a modern networking platform, connected to a professional laboratory and medical institutions, and delivered results anonymously to achieve the first 90 of 90-90-90 target.

## Supporting information

S1 File Data The number of times search results of 70 HIV-1-positive urine sample participants. (XLSX)

S2 File Data The number of times search results of 360 HIV-1-negative urine sample participants. (XLSX)

S3 File Data Background information of 70 HIV-1-positive individuals. (XLSX)
